# Authors' Response to Peer Reviews of “Effects of Pharmacogenomic Testing in Clinical Pain Management: Retrospective Study”

**DOI:** 10.2196/37242

**Published:** 2022-05-03

**Authors:** Christian Tagwerker, Mary Jane Carias-Marines, David J Smith

**Affiliations:** 1 Alcala Testing and Analysis Services San Diego, CA United States

**Keywords:** pharmacogenomics, pain management, drug-drug interaction, DDI, pharmacy, prescriptions, genetics, genomics, drug-gene interaction, pain

*This is the authors’ response to peer-review reports for “Effects of Pharmacogenomic Testing in Clinical Pain Management: Retrospective Study.”*

## Round 1 Review

### Reviewer AI [1]

#### General Comments

Authors of this manuscript [2] have determined the impact of pharmacogenetic (PGx) testing on pain medication prescribing. A retrospective analysis was conducted with 171 patients in a pain management clinic during 2016 to 2018 within the western United States. A novel deep sequencing (>1000X) PGx panel is described encompassing 23 genes combined with PGx dosing guidance, drug-gene interaction (DGI), and drug-drug interaction (DDI) reporting to prevent adverse drug reaction (ADR) events. This manuscript is interesting and well-written. However, the Methods and Discussion section of the manuscript could be improved for clarity. Please refer to my comments below.

#### Specific Comments

##### Major Comments

###### Abstract

1. What was the primary outcome of this study? Is it to report the number of cases where PGx information could be used to optimize drug dosing?

Response: Correct, but also to summarize the type of drugs altered and scenarios in a clinic that had never utilized PGx reports before, as much as possible based on urine drug toxicology (UDT) data and with limited access to patient progress notes, to better describe this:

Abstract: Objectives section was changed to “The following study summarizes an extended pharmacogenomic (PGx) sequencing panel intended for medication dosing and prescription guidance newly adopted in a pain management setting. The primary outcome of this retrospective study reports the number of cases and types of drugs covered, for which PGx data appears to have assisted in optimal drug prescription and dosing.”

The introduction sentence was changed to “The aim of this study is to evaluate the overall utilization and describe how PGx report recommendations (including genetic based dosing guidance (PGx), drug-gene interaction (DGI) and drug-drug interaction (DDI) based guidance) were applied to optimize drug dosing in a clinical setting which had not previously relied on pharmacogenetic test reports. Changes in prescription, patient compliance and drug usage were monitored based on updated medication lists and data in associated quantitative urine drug toxicology (UDT) reports, with limited access to patient progress reports.”

2. “This study demonstrates a successful implementation of PGx testing utilizing an extended PGx panel combined with a customized, informational report to help improve clinical outcomes.” Did authors develop a software platform to generate a customized, informational report to help improve clinical outcomes? I do not see any discussion on this matter.

Response: Yes, this may have not been described enough but was mentioned in the Methods section 2.6; the following has been added to section 2.6:

“Specifically, to accommodate reporting based on 23 genes, 141 SNPs or indels, and associated haplotypes newly combined in this panel (Supplementary Table 1), TSI bioinformaticians collaborated with Alcala Testing and Analysis Services (ATAS) scientists to include the most up-to-date guidance across 2 evidence levels for PGx dosing and drug-drug interactions (DDI) (Fig. 2). Recommendations from six different international pharmacogenetic consortia, professional societies or regulatory bodies (Clinical Pharmacogenetics Implementation Consortium - CPIC, Dutch Pharmacogenetics Working Group - DPWG, US Food and Drug Administration - FDA, European Medicines Agency - EMA, Canadian Pharmacogenomics Network for Drug Safety - CPNDA, American College of Medical Genetics and Genomics - ACMG) were incorporated in the reporting algorithm. The updated recommendations covered 13 drug categories and 198 drugs with a major emphasis on Pain, Psychiatry and Addiction Medicine drugs (Supplementary Table 3).”

What were the parameters of the effectiveness and safety of treatment in evaluated patients? Did you do any statistical testing to find an association between the presence of a polymorphic gene variant and the impact of pharmacotherapy? Did you have a control group?

Response: Changes in prescription, patient compliance, and drug use were monitored based on updated medication lists and data in associated quantitative UDT reports, with limited access to patient progress reports. Therefore, the effectiveness and safety of treatment could not be established through progress notes and on a limited basis for the 3 case studies (assuming routine clinical practice in a pain management setting). UDT reports were the primary source of information to monitor and evaluate if changes in prescriptions were made and if recommendations of the PGx report were followed. The evaluation could only focus on the PGx report recommendation given by consortia guidelines and could not determine prescription changes based off of polymorphic gene variants themselves. There was no control group to evaluate as this was data focused on patients (N=171) from one pain management clinic only.

###### Introduction

3. I would be interested in having a brief introduction to currently available PGx panels, and what the strengths of the panel in this study are.

Response: An excellent review of currently available PGx panels as of 2018 has been summarized in the Introduction section as follows: “In 2018, Fabbri et al. described 38 commercially available PGx test panels offering personalized medication prescription guidance in clinical settings. The only genes included in all of these panels are CYP2D6 and CYP2C19. Thirty-one out of the 38 panels (82%) include 8 genes or less (15). PGx testing as described in this study encompasses deep sequencing (>1000X) of 141 SNPs or indels across 23 genes by Next-Generation Sequencing.”

###### Methods

4. “23 genes were selected based on having the most clinical utility in PGx at the time of design in April 2016 (ADRA2A, CES1, COMT, CYP1A2, CYP2C19, CYP2C9, CYP2D6, CYP3A4, CYP3A5, DRD1, DRD2, F2, F5, GNB3, HTR1A, HTR2A, HTR2C, MTHFR, OPRM1, SLC6A2, SCL6A4, SLCO1B1, VKORC1).” What were the criteria used to narrow down genes that authors considered of most clinical utility in PGx?

Response: Updated this paragraph to reference the process with Translational Software Inc (TSI) to select genes and haplotypes as updated in section 2.6: “23 genes were included in the described PGx panel at the time of design in April 2016 (ADRA2A, CES1, COMT, CYP1A2, CYP2C19, CYP2C9, CYP2D6, CYP3A4, CYP3A5, DRD1, DRD2, F2, F5, GNB3, HTR1A, HTR2A, HTR2C, MTHFR, OPRM1, SLC6A2, SCL6A4, SLCO1B1, VKORC1), to include the most up-to-date guidance covering 198 drugs with a major emphasis on Pain, Psychiatry and Addiction Medicine as described below in section 2.6.”

5. “75 target regions were covered by 82 amplicons with an average amplicon size of 250 base pairs (bp)” Can you elaborate on 75 target regions? Did the authors have multiple target regions per gene? If so, details should be provided.

Response: Elaborated upon in changes in section 2.2, 75 is a typo, should be 79: “Unique reference single-nucleotide polymorphism cluster ID (rsID) numbers were assigned per target coordinate and region. 79 target regions (defined across Start and Stop coordinates, see Supplementary Table 1) covering 141 SNPs or indels were covered by 82 amplicons with an average amplicon size of 250 basepairs (bp) across 23 genes. Multiple target regions covering multiple rsIDs were targeted across each gene (e.g. 27 rsIDs within CYP2D6, see Supplementary Table 1).”

6. What were the medical conditions of patients with pain management in this study? Was it varied across patients in this cohort? I would like to see the authors’ discussion on this.

Response: As previously described the full treatment history of outcomes, effectiveness, and safety of treatment could not be established through progress notes and only on a limited basis for the 3 case studies. The medical condition of each patient could not be established, as these were “snapshots” of patients’ prescriptions during treatment based on UDT reports being the primary source of information to monitor and evaluate if changes in prescriptions were made and if recommendations of the PGx report were followed. A further result and discussion section on this would also exceed the length and focus of this manuscript.

7. “PGx reporting were obtained retrospectively from patients (n=171) in a pain management clinic representing an ethnically diverse patient population from 2016 to 2018 within the western United States.” Although authors report that they have an ethnically diverse patient population, no descriptive statistics on demographics, age, and clinical information was provided.

Response: Essentially correct, the population within the western United States and San Diego area was assumed to be relatively ethnically diverse; while the age of patients was available, there were no demographic data available for this San Diego cohort (SDC) patient population from 2016 to 2018. However, Table 2 compared the SDC to 5 super populations from the 1000 Genomes Database. Additional statistics were now performed by the authors (see new Supplementary Table 6) to better characterize the SDC to the 1000 Genomes Database frequencies; therefore, the sentence in the Methods on page 4 has been changed to “...representing a patient population from 2016 to 2018 within the western United States. While no patient demographics data was available, Table 2 shows the genotype frequencies of the ‘SDC’ cohort of this study compared to 5 super populations from the 1000 Genomes Database: African (AFR), South Asian (SAS), Ad Mixed American (AMR), East Asian (EAS) and European (EUR). Pearson’s correlation analysis (Supplementary Table 6) showed the ‘SDC’ cohort positively correlates to all allele frequencies in the 1000 Genomes Database (ALL=0.76, *P*=1.019 x 10-11). SDC cohort (n=171) closely correlates to the Ad Mixed American (AMR=0.77), European (EUR=0.78) and South Asian (SAS=0.78) super populations but is less representative of the East Asian (EAS=0.54) and African (AFR=0.55) population frequencies.” Also see note in point 11 below.

8. What factors were tested on urine toxicology and progress report?

Response: Added “see sections 2.6 and 2.7” in Methods introduction paragraph. As per reviewers point 2 above, section 2.6 was elaborated on by adding: “Specifically, to accommodate reporting based on 23 genes, 141 SNPs or indels, and associated haplotypes newly combined in this panel (Supplementary Table 1), TSI bioinformaticians collaborated with Alcala Testing and Analysis Services (ATAS) scientists to include the most up-to-date guidance across 2 evidence levels for PGx dosing and drug-drug interactions (DDI) (Fig. 2). Recommendations from six different international pharmacogenetic consortia, professional societies or regulatory bodies (Clinical Pharmacogenetics Implementation Consortium - CPIC, Dutch Pharmacogenetics Working Group - DPWG, US Food and Drug Administration - FDA, European Medicines Agency - EMA, Canadian Pharmacogenomics Network for Drug Safety - CPNDA, American College of Medical Genetics and Genomics - ACMG) were incorporated in the reporting algorithm. Integrated recommendations covered 13 drug categories and 198 drugs with a major emphasis on Pain, Psychiatry and Addiction Medicine drugs (Supplementary Table 3).”

Section 2.7 specifies details on the drug adherence testing

“Urine toxicology reports reviewed by clinical laboratory scientists with ASCENT™ review software (IndigoBio Automation, Carmel, IN) (21) were made available by routine HPLC-MS/MS presumptive and confirmatory urine drug testing at ATAS from 2016-2018 (22).”

####### Results and Discussion

9. While the manuscript describes 3 patients (patient A, B, and C) who did not stick to the treatment regimen and drug response adversaries, did patients who stuck to treatment regimens based on PGx testing show any side effects or did they do any survey for reporting pain symptoms? For example, were they tested for ADRs or partial or complete response to treatments?

Response: As described above, the full treatments themselves and side effects could not be established through progress notes and only on a limited basis for the 3 case studies. The medical condition of all patients could not be established, as these were “snapshots” of patients’ prescriptions during treatment based on UDT reports being the primary source of information to monitor and evaluate if changes in prescriptions were made and if recommendations of the PGx report were followed. A further Results and Discussion section on this would also exceed the length and focus of this manuscript.

10. I would like to see a discussion on key limitations of this study and further improvement on this study.

Response: Good point, the following paragraph has been added to the Discussion section: “Limitations within the retrospective study presented here include lack of detailed patient demographics associated with UDT and PGx reports, limited access to progress notes and long-term treatment outcomes. Rather than resorting to 1000 Genome Database population frequencies to characterize the SDC cohort, specific demographics and additional case studies as the three presented above would allow more comprehensive insights as to the combinatorial effect of prescription drugs among polypharmacy, pain management patients.”

11. Have you looked into genotype frequencies of different ethnic populations in your study? What benefits do you anticipate by studying PGx-guided treatment interventions on diverse ethnic populations?

Response: As there are no descriptive statistics on demographics for this particular patient population from 2016 to 2018 and we changed the sentence to “...representing a patient population from 2016 to 2018 within the western United States...,” this is a good point, and Table 2 does at least provide an overview of population frequencies of relevant genotypes across 5 super populations (1000 Genomes Project). We think studying PGx-guided treatment interventions on diverse super populations show all populations are possibly affected for these serious ADRs, albeit at less frequency for certain metabolizer types. The authors have added more description in the Results section on page 8 as to how serious ADRs caused by PGx guidance based on only the genotype have been observed in this study (see Figure 3) affecting mainly CYP2D6 and CYP2C19 poor or rapid metabolizer types: “Phenotypes and associated genotypes were summarized in Table 2 with an overview of population frequencies compared to this ‘SDC’ cohort. As shown in Figure 3, 5.5% of 146 patients showed serious adverse drug reactions (ADRs) based on changes in either CYP2C19 (Poor, Intermediate to Rapid metabolizers), CYP2D6 (Poor or Ultra-Rapid Metabolizers) and one SLCO1B1 reduced function genotype. CYP2C19 genotype frequencies for 3 metabolizer types causing serious ADRs are spread across all 5 super populations ranging from 0.9 to 47.4% frequency (Table 2, CYP2C19 section). CYP2D6 genotype frequencies for Intermediate to Ultra-Rapid Metabolizers range from 1.2 to 57.1% frequency and SLCO1B1 Poor Function genotypes from 1.8 to 37% (Table 2, CYP2D6 and SLCO1B1 section). While South Asian (EAS) population frequencies for CYP2C19 Ultra-Rapid Metabolizers and CYP2D6 Poor Metabolizers are determined as non-existent in the 1000 Genome Database data, more recent studies show frequencies of 0.24% (23) and 0.84% (24) respectively, indicating possible occurrence within the EAS super population.”

And in the Discussion section on page 11:

“Serious ADRs can occur based on incidences of these metabolizer types in all 5 super populations for prescriptions such as amitriptyline, citalopram or clopidogrel, metoprolol, paroxetine, simvastatin and tramadol.”

###### Conclusion

12. “This study demonstrates the predictive value of PGx testing combined with a customized informational report to help improve clinical outcomes, which resulted in increased utilization on patients in a pain management setting.” On what basis do the authors claim increased utilization on patients in a pain management setting? Did you do any statistical analysis to back up this statement?

Response: As described in the changes to the Abstract and Introduction, the clinical setting described had previously not relied on PGx testing and reports, and therefore, the utilization was studied, which showed changes in prescriptions based on PGx report recommendations. “Increased utilization” may have been the wrong wording, rather “successful application”; the discussion sentence was changed to:

“In summary, the effect of PGx reports newly made available to medical staff in this context seems quite significant as observed by the individual PGx dosing/metabolizer status, DGI and DDI recommendations showing a corresponding modification of the medication regimen for each patient. Preventative action was observed for all serious interactions and only moderate interactions were tolerated where there may not have been other alternatives. This study demonstrates the predictive value of PGx testing combined with a customized informational report to help improve clinical outcomes, which resulted in successful application on patients in a pain management setting.”

### Reviewer CK [3]

#### General Comments

This paper touches a very important and clinically relevant issue of adverse drug interactions with genetic variations and how these variants affect the patient’s response to the specific drug. It focuses on utilizing pharmacogenetic (PGx) testing in clinical practice, which takes into account these relevant drug-genome interactions when prescribing drug therapy. They appropriately chose an acceptable sample size >150 and follow them for a significant period of time (>18 months). Importantly, they have performed retrospective studies, which makes a good case for the utility of PGx testing. They also lay a good background on what other technologies for PGx testing are being routinely used in current clinical settings.

#### Specific Comments

##### Major Comments

I have no negative comments for this paper; here are some positive comments:

1. I especially find it very impressive that various figures and tables were added to the paper, which shows their thorough work. Figure 1 clearly describes PGx testing compared to UDT reports. Figure 2 indicates the potential drug-gene and drug-drug interactions as provided by the PGx testing and suggests alternatives in case of serious and moderate interactions based on information from various regulatory bodies. Tables 1 and 2 are of significant interest because they focus on genotype, phenotype, and population frequencies for the genes in the panel. Figure 3 focuses on the importance of PGx testing in identifying moderate to serious drug-drug or DGIs.

Overall, I find this study very impactful especially with the advent of individualized drug therapy and targeted drug recommendations.

2. The results and discussion focus on how recommendations and dosage were changed based on PGx reports and resulted in favorable outcomes for the patients. This shows the utility of PGx in areas where health care professionals are not aware of these interferences or interactions between drug-gene and drug-drug.

3. I am not sure how many clinically relevant genes have changed or updated since April 2016, but this paper lays the groundwork for a more up-to-date gene panel to be used. I would be interested in seeing the outcome with a more up-to-date gene list but that does not necessarily have to be addressed in this paper.

##### Minor Comments

4. This was a very legibly worded paper, and I found no issues with the English or the scientific language that was used.

## Abbreviations

ADR: adverse drug reaction
AFR: African
AMR: Ad Mixed American
ATAS: Alcala Testing and Analysis Services
bp: basepairs
DDI: drug-drug interaction
DGI: drug-gene interaction
EAS: East Asian
EUR: European
PGx: pharmacogenomic
rsID: reference single-nucleotide polymorphism cluster ID
SAS: South Asian
SDC: San Diego cohort
TSI: Translational Software Inc
UDT: urine drug toxicology

## References

[1] Archchilage M (2022). Peer review of "Effects of Pharmacogenomic Testing in Clinical Pain Management: Retrospective Study". JMIRx Med.

[2] Tagwerker C, Carias-Marines MJ, Smith DJ (2022). Effects of Pharmacogenomic Testing in Clinical Pain Management: Retrospective Study. JMIRx Med.

[3] Rathi K (2022). Peer review of "Effects of Pharmacogenomic Testing in Clinical Pain Management: Retrospective Study". JMIRx Med.

